# TRPV1 channels as a newly identified target for vitamin D

**DOI:** 10.1080/19336950.2021.1905248

**Published:** 2021-04-07

**Authors:** Wentong Long, Janyne Johnson, Subha Kalyaanamoorthy, Peter Light

**Affiliations:** a Department of Pharmacology and the Alberta Diabetes Institute, University of Alberta, Edmonton, Canada; b Department of Chemistry, University of Waterloo, Waterloo, Canada

**Keywords:** Vitamin D, 25-hydroxyvitamin D, 25OHD, TRPV1, TRP channels

## Abstract

Vitamin D is known to elicit many biological effects in diverse tissue types and is thought to act almost exclusively upon its canonical receptor within the nucleus, leading to gene transcriptional changes and the subsequent cellular response. However, not all the observed effects of vitamin D can be attributed to this sole mechanism, and other cellular targets likely exist but remain to be identified. Our recent discovery that vitamin D is a partial agonist of the Transient Receptor Potential Vanilloid family 1 (TRPV1) channel may provide new insights as to how this important vitamin exerts its biological effects either independently or in addition to the nuclear vitamin D receptor. In this review, we discuss the literature surrounding this apparent discrepancy in vitamin D signaling and compare vitamin D with known TRPV1 ligands with respect to their binding to TRPV1. Furthermore, we provide evidence supporting the notion that this novel vitamin D/TRPV1 axis may explain some of the beneficial actions of this vitamin in disease states where TRPV1 expression and vitamin D deficiency are known to overlap. Finally, we discuss whether vitamin D may also act on other members of the TRP family of ion channels.

## Introduction

Vitamin D is a lipophilic hormone that is essential in regulating calcium transport processes in many different tissues. Calcifediol (25-hydroxy vitamin D or 25OHD) and calcitriol (1,25-hydroxy vitamin D or 1,25OHD) are the major circulating forms of vitamin D present in the human body. Clinically, vitamin D levels are a measurement of circulating 25OHD in blood, with concentrations >70 nmol being considered optimal [1,2]. In addition to rickets, vitamin D deficiency is associated with many inflammatory and autoimmune diseases and neurological disorders, including arthritis [3,4], psoriasis [5], multiple sclerosis [6,7], Alzheimer’s disease, and Parkinson’s disease [8]. Last year, vitamin D became a popular yet controversial topic since low levels of 25OHD are correlated with severe complications and increased mortality observed in the SARS-CoV-2/COVID-19 pandemic [9,10]. Studies suggest that adequate levels of vitamin D may prevent the initiation of a “cytokine storm” by suppressing the hyper-activation of the adaptive immune system and enhancing the innate immune system’s response to viral load [10–12]. Vitamin D is generally considered eliciting its effects by activating the nuclear vitamin D receptor (VDR), resulting in gene transcriptional changes that alter cellular behavior. However, a number of cellular effects have been observed that cannot be fully accounted for by the exclusive actions of vitamin D on the VDR alone. For example, VDR is not expressed in naive T-cells [13–15]. Therefore, alternative molecular targets that underlie the effects of vitamin D likely exist, but these targets remain to be elucidated.

With respect to vitamin D and ion channel function, our laboratory has recently provided strong evidence that vitamin D can also directly act on the Transient Receptor Potential (TRP) Vanilloid family 1 (TRPV1) channel, a member of the TRP superfamily of ion channels [16]. Vitamin D has been shown to negatively regulate N-methyl-D-aspartate (NMDA) receptors, kainate receptors, and neuronal L-type voltage-sensitive calcium channels and may therefore confer neuroprotection [17,18]. Our observations suggest that TRPV1, and perhaps other TRP channels, may be novel cell-surface receptors for the biological actions of vitamin D. TRP channels are a family of nonselective cation channels that are distributed in most tissues throughout the body, and their activity can be modulated by environmental stimuli such as temperature, taste, pH, light, and nociception that regulate intracellular calcium levels and membrane excitability in many tissues [19–21]. Due to their crucial roles in diverse tissue types, TRP channels have attracted significant pharmaceutical interest [22–26] .

TRPV1 is a calcium-selective channel that is activated by heat, low pH, and various endogenous and exogenous agonists [27]. Interestingly, TRPV1 is expressed in various tissues throughout the human body, including neurons, immune T-cells, nociceptive C fibers that innervate airways, and airway epithelial cells [24,28]. Therefore, TRPV1 channels may mediate some of the biological actions of vitamin D that cannot be explained by its actions on the nuclear VDR alone [29]. In this review, we discuss the historical evidence for the actions of vitamin D, the mechanisms by which it may regulate TRPV1 channel activity, and the evidence for other TRP channel family members being potential targets for vitamin D.

## The discovery of vitamin D

Initially sourced from cod liver oil, vitamin D’s discovery represented a breakthrough in the treatment of rickets, a disease of impaired bone calcification [30–32]. Further research revealed that children who suffered from rickets could also be cured by exposure to artificial ultraviolet (UV) light or summer sunlight [31,33]. This led to the concept that UV light induces vitamin D synthesis [34]. Vitamin D2 and D3 were subsequently isolated and then identified as the major molecules that carry these antirachitic properties [35,36], confirming rickets as a disease of vitamin D deficiency resulting in hypocalcification of bone. Interestingly, bones from patients with rickets could still calcify if sufficient calcium and phosphate were provided in the diet [32,37,38]. Therefore, it was concluded that vitamin D deficiency results in impaired calcium absorption and active transport of calcium required for bone mineralization [39,40], indicating that vitamin D directly facilitates intestinal calcium absorption. However, the precise cellular mechanism by which vitamin D induces active calcium transport still remains a subject of debate.

## Vitamin D, calcium transport, and unanswered questions

Early studies investigated the underlying cellular mechanisms by which vitamin D facilitates calcium transport from the intestinal lumen to the bloodstream. It was initially proposed that vitamin D might act as a calcium carrier, where calcium and vitamin D form a complex that is then absorbed into the blood [41]. However, subsequent research determined that vitamin D must be metabolized into 25OHD and 1,25OHD within the body after absorption to become biologically active. Thus, this theory was insufficient to fully explain the link between vitamin D and intestinal calcium transport. A second concept was introduced whereby vitamin D alters passive calcium permeability via a paracellular mechanism [42] as vitamin D increases paracellular calcium flux in both directions [43–45], via alteration in gap-junctional permeability [46,47]. Although the precise mechanisms by which calcium permeability is altered by vitamin D still remain unclear, a third theory suggests that vitamin D can increase active calcium transport across the intestinal membrane [45,48]. Collectively, these concepts indicated that vitamin D regulates endogenous calcium transport processes in the intestine [40,41,49]. However, precisely how this occurs remained to be elucidated and was partially explained by the subsequent discovery of the VDR.

## In search of the VDR

The discovery of the canonical nuclear VDR can be traced back to early observations of animal models of rickets treated with a high dose of vitamin D. These animals could achieve an optimum calcium absorption in 3–5 h. In contrast, animals treated with a lower dose of vitamin D (100 IU) required 12–15 h to reach maximum calcium absorption [50,51]. This notable time lag between the oral vitamin D administration and the maximum enhancement of calcium absorption suggested that vitamin D either induced the expression of crucial transport enzymes or altered plasma membrane structures necessary for calcium absorption. Subsequent experiments revealed that vitamin D promotes protein synthesis in the mucosal cells of the intestine [50–53], and the VDR was located in the nuclei of enterocytes [54–56]. By the late 1980s, the complete human VDR cDNA was cloned and expressed [57]. Many of the observed actions of vitamin D are generally considered occurring through an interaction between the nuclear VDR and the retinoid X receptor that initiates downstream gene transcription and protein synthesis to elicit the cellular response [58,59].

Although the now-established classical 1,25OHD/VDR signaling axis can reasonably explain the longer-term changes in intestinal calcium absorption, it still cannot fully explain the phenomenon of the rapid calcium flux induced by vitamin D. In this regard, it has been shown that 1,25OHD increases calcium uptake within 30 min of administration in an *ex vivo* intestinal perfusion model [60]. Furthermore, 1,25OHD induces acute calcium uptake into intestinal lysosomes or isolated enterocytes within 10–20 min of administration [61,62]. These findings imply that other as yet unidentified targets for vitamin D may exist, as the classical 1,25OHD/VDR signaling axis involves gene transcriptional changes that require many hours to manifest. Therefore, it is plausible that vitamin D may directly act on ion channels/transporters, explaining the observed rapid changes in calcium flux. This concept is supported by the observation that 25OHD binds to the plasma membrane, implying that 25OHD has the potential to localize and interact with membrane proteins [56].

## TRPV1 as a novel VDR

In addition to vitamin D’s classical role in regulating calcium absorption, more recent studies have confirmed that this vitamin also possesses a direct regulatory role in the human immune system [63,64]. In this regard, vitamin D deficiency is associated with autoimmune diseases such as inflammatory bowel disease, type 1 diabetes, and multiple sclerosis [6,7,65,66], with 1,25OHD inhibiting the progression of these diseases [3,67,68]. Interestingly, very recent research suggests that vitamin D deficiency is associated with severe symptoms and a high fatality rate in COVID-19 patients [10], with sufficient vitamin D levels required to oppose the initiation of an immune cell-mediated cytokine storm [11]. It is thought that vitamin D may dampen the over-reactive Th1/CD4+ immune cells by reducing their production of pro-inflammatory cytokines such as interferon gamma (INFγ), IL17, and tumor necrosis factor alpha (TNFα) [69,70]. Of note is the fact that naive T-cells do not express any VDRs, indicating that vitamin D may act through additional, as yet undiscovered, pathways to regulate cellular responses in the immune system.

While the classical 1,25OHD/VDR axis underlies many of the observed effects of vitamin D, the evidence presented above indicates that additional cellular targets may mediate some of the documented effects of this vitamin. In this regard, our group recently discovered that TRPV1 channel activity is regulated by physiological nanomolar concentrations of both 25OHD and 1,25OHD, where they act as partial agonists of TRPV1 [16].

The potential importance of this discovery is supported by the substantial overlap in the disease profiles associated with vitamin deficiency and the expression profile of TRPV1. These include metabolic [65,71] and nociceptive disorders [72,73], inflammation, autoimmune disease [74,75], multiple sclerosis [7,76], Parkinson’s disease [8,77,78], psoriasis [79,80], and inflammatory lung disease [81,82]. With respect to the immune system, TRPV1-mediated calcium influx is a key step required for CD4+ T-cell activation and proliferation [83]. In our recent study, we observed that physiological concentrations of either 25OHD or 1,25OHD significantly reduce TNFα and INFγ production resulting from the T-cell receptor (TCR) activation of CD4+ T-cells [16]. These results suggest that 25OHD and 1,25OHD may downregulate the activation of naive T-cells via a VDR-independent mechanism by suppressing TRPV1 activity. Since anti-CD3 and anti-CD28 were used to activate the TCR pathway, we suggested that 25OHD and 1,25OHD may reduce the protein kinase C (PKC)-mediated potentiation of TRPV1, reducing cytokine production [84,85].

Numerous studies indicate that TRPV1 is related to neuropathic pain caused by inflammatory disease and cancer [86]. Of note, vitamin D deficiency is also associated with chronic pain experienced in cancer and metabolic diseases and may contribute to its progression [87,88]. Surprisingly, topical application of capsaicin, a full TRPV1 agonist, or vitamin D demonstrates promising results in relief of neuropathic pain [89,90]. This may seem counterintuitive although excessive activation of nociceptive neurons with capsaicin is thought to lead to chronic desensitization of TRPV1 and a subsequent reduction in neuronal activity. Of direct relevance to nociceptive disorders, our recent results demonstrated that 25OHD acutely reduced the capsaicin-induced calcium influx into trigeminal neurons within minutes of application. This finding also provides further evidence that the mechanism involved is independent of its actions on the nuclear VDR and does not involve transcriptional regulation in this system. Taken together, our findings support the concept that vitamin D may act directly on TPRV1 to modulate channel activity and therefore be a potentially new mechanism involved in the regulation of nociceptive neuronal activity.

## Vitamin D as an endogenous partial agonist of TRPV1

TRPV1 acts as a transducer of chemical and thermal stimuli such as heat and acidity, as well as responding to endogenous and exogenous ligands [19–21]. TRPV1 was first identified as the cellular receptor for capsaicin, the major active compound found in hot chili peppers [73,91]. Since this discovery, a number of endogenous lipophilic compounds have been shown to regulate TRPV1 activity. These include oxidized linoleic acid metabolites [92,93], *N*-acylethanolamines and *N*-acyldopamines (e.g. oleoyl dopamine, OLDA) [94,95], anandamide [96], phosphoinositides [97,98] and long-chain acyl CoA esters [97,99]. Many of these endogenous ligands likely play a role in mediating inflammatory hyperalgesia and thermal allodynia [94,95,100–102] (Table 1). It is worth noting that most of these ligands are full TRPV1 agonists or modulators, whereas there are very few examples of antagonists or partial agonists that may negatively regulate TRPV1 activity, provide basal TRPV1 activity, or oppose the actions of full agonists.

**Table 1.** A list of identified agonists, partial agonists, and modulators of TRPV1

Name and type of agonist [full agonist, partial agonist, or modulator]	TRPV1 overexpression systems		Biological effects	PKC potentiation effects	*In silico* docking	Source of agonist
	Direct activation of TRPV1	Effect on capsaicin/low-pH–induced TRPV1 activities				
Capsaicin [full agonist]	Yes, EC_50_ = 34 nM [94], 26 nM [125]; EC_50_ = 0.23 µM [92] in HEK293 cells	ND	Produces thermal hyperalgesia in rodents [27,94]	Potentiates capsaicin-induced TRPV1 activities [106,107]	Vanilloid-binding pocket in the “head-down, tail -up” configuration [126]	Exogenous compound from hot chili peppers [27]
Resiniferatoxin (RTX) [full agonist]	Yes, EC_50_ = 0.15–0.2 nM [127,128]	Reduces capsaicin-induced TRPV1 activity [127]	Produces transient or chronic thermal hyperalgesia in rodents [129]	Activates PKC [130] and reduces PKC-potentiated capsaicin-induced TRPV1 activities [127]	Vanilloid-binding pocket [91,131]	Tricyclic diterpene from the Moroccan cactus, *Euphorbia resinifera* [132]
25-OHD and 1,25OHD [partial agonist]	Yes, 100 nM 25OHD or 1,25OHD induces TRPV1-mediated currents [16]	Reduces capsaicin-induced sustained currents [16]	Reduces capsaicin-induced current in TRG; reduces T-cell activation and cytokine production [16]	Inhibits PKC potentiation requiring S502 [16]	Vanilloid-binding pocket, perpendicular to capsaicin-binding position [16]	UVB-mediated synthesis, hepatic and renal metabolism
N-Arachidonoyl-ethanolamine (anandamide, AEA) [partial agonist]	Yes, EC_50_ = 5.3 µM [103], EC_50_ = 0.55 µM [125]	Reduces effects of capsaicin but increases low-pH–induced calcium influx in DRG [104,133]	Induces arterial relaxation [103]	Potentiates AEA-induced TRPV1 activity [134]	Binds in regions formed by S1–S4 in TRPV1, with head group interacting with Y554 [135]	CNS [103] and macrophages [136]
N-arachidonoyl-dopamine (NADA) [full agonist]	Yes, EC_50_ = 63 nM [94]; ≈50 nM [125]	Alters capsaicin-induced desensitization of TRPV1 currents [125]	Increases intracellular calcium in rat DRG [125]; produces thermal hyperalgesia in rodents [94]	Potentiates effects of NADA. Requires both S502 and S800 [137]	ND	CNS: striatum, hippocampus, cerebellum, and dorsal root ganglia [138]
N-oleoyldopamine (OLDA) [full agonist]	Yes, EC_50_ = 36 nM [94] in HEK293 cells	ND	Produces thermal hyperalgesia in rodents [94]	Potentiation of OLDA-induced TRPV1 activity [101]	ND	CNS: striatum, hippocampus, cerebellum, and dorsal root ganglia [138]
N-oleoyl-ethanolamide (OEA) [full agonist]	No measurable ^45^Ca^2+^ uptake by OEA in HT5-1 cells [139]. No measurable current in *Xenopus* oocytes [140]	Reduces capsaicin- induced ^45^Ca^2+^. [139] Enhances low-pH–induced TRPV1 currents [140]	Increases DRG Ca^2+^ influx in a PKC-dependent manner [140]	Potentiates OEA-induced TRPV1 activity [140]	ND	Gastrointestinal tract [141]
2-Arachidonoyl-glycerol (2AG) [full agonist]	Yes, EC_50_ = 0.85 µM in HEK293 cells [142,143]	Reduces capsaicin- induced Ca^2+-^influx [142]	Induces vasorelaxation in rat mesenteric arteries [143]	PKC inhibition does not affect 2-AG-induced TRPV1 activity [143]	ND	2-AG is a metabolite of diacylglycerol and biosynthesized in the DRG [143]
20(S)-HETE [full agonist]	Yes, EC_50_ = 12 µM in HEK293 cells [92]	Rescues capsaicin- and low-pH–induced TRPV1 current desensitization [92]	10 µM 20-HETE induces currents and Ca^2+^ influx in mouse DRG neurons [92]	PKC and PKA inhibitors reduce 20-HETE-induced TRPV1 activities [92]	ND	Arachidonic acid metabolite [92]
12(S)-HpETE [full agonist]	Yes, EC_50__ _= 8 µM in HEK293 cells [93]	ND	ND	ND	ND	Initially found in platelets [144]
15(S)-HpETE [full agonist]	Yes, EC_50__ _= 8.7 µM in HEK293 cells [93]	ND	ND	ND	ND	Airway epithelial cells, eosinophils, blood vessels, and reticulocytes [144]
5(S)-HpETE [full agonist]	Yes, EC_50_ = 9.2 µM in HEK293 cells [93]	ND	ND	ND	ND	Neutrophils [145]
Phospho-inositide PtdIns(4)P (PIP) [modulator]	Yes, EC_50_ = 4.9 µM (+100 mV)/11.1 µM (−100 mV) in HEK293 cells [98]	Reduces capsaicin-induced desensitization of TRPV1 currents [98]	Increases Ca^2+^ influx in DRG leads to depletion of PIP_2_ [146]	ND	ND	Phospholipid component of cell membranes [146,147]
Phospho-inositide PtdIns(4,5)P2 (PIP2) [modulator]	Yes, EC_50_ = 32.4 µM (+100 mV)/70.2 µM (−100 mV) in HEK293 cells [98]	Reduces capsaicin-induced desensitization of TRPV1currents [98]	Increases Ca Ca^2+^ influx in DRG, leading to depletion of PIP_2_ [146]	ND	ND	Lipid component of cell membrane [146,147]
Long-chain acyl CoA esters (LC-CoAs) [modulator]	No, LC-CoAs do not activate TRPV1 directly but potentiate capsaicin-induced TRPV1 activity and rescue desensitization. [99]	Rescues capsaicin- and low pH desensitization of TRPV1 currents in [99]	ND	ND	Predicted to interact with R702 and K711 on the TRP domains adjacent to the cytoplasmic membrane interface [99]	Ubiquitous [148,149]

ND: not determined; EC50: concentration that activated 50% of the maximum TRPV1 activity; agonists, partial agonists and modulators of TRPV1 .

Our recent research has now identified the vitamin D metabolites 25OHD and 1,25OHD as endogenous partial agonists of TRPV1 [16]. The application of physiological nanomolar concentrations of 25OHD or 1,25OHD alone evoked small but measurable TRPV1 currents that were ~10% of the capsaicin- or OLDA-induced currents, but unlike full agonists such as capsaicin, these small currents did not desensitize. Conversely, the TRPV1 stimulatory effects of the full agonists capsaicin and OLDA were reduced in the presence of 25OHD or 1,25OHD, indicating that these vitamin D metabolites are partial agonists of TRPV1. To further elucidate the mechanism by which 25OHD modulates TRPV1, our group studied TRPV1 single-channel activity TRPV1 in the presence of 25OHD, as this vitamin D metabolite is the most common circulating form of this vitamin. We found that 25OHD augments TRPV1 open probability by stabilizing the open state and increasing the frequency of opening events. 25OHD also reduced TRPV1 open probability in the presence of the full agonist capsaicin. These results further confirm that 25OHD is a partial agonist of TRPV1 and, therefore, can oppose the actions of endogenous full agonists of TRPV1.

## Where does 25OHD bind to TRPV1?

Previous research on endogenous TRPV1 ligands might provide clues as to how and where 25OHD acts upon TRPV1. For example, the endogenous cannabinoid anandamide is recognized as a low-affinity agonist of TRPV1 [96,103]. In trigeminal neurons, the application of anandamide can significantly reduce capsaicin-induced TRPV1 currents. Anandamide can also augment the low-pH–induced calcium uptake mediated by TRPV1 [104]. In contrast, we demonstrated that 25OHD did not alter the low-pH–induced TRPV1 activity. Therefore, 25OHD does not seem to act in the same manner as anandamide and may interact with TRPV1 via a different mechanism.

**Figure 1.**
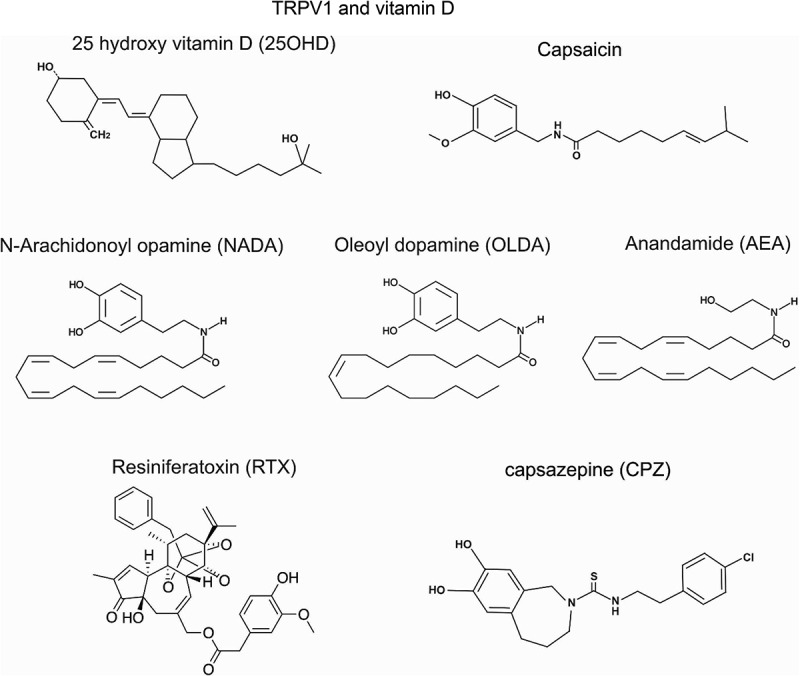
Structures of several known TRPV1 ligands compared to the structure of 25OHD

**Figure 2.**
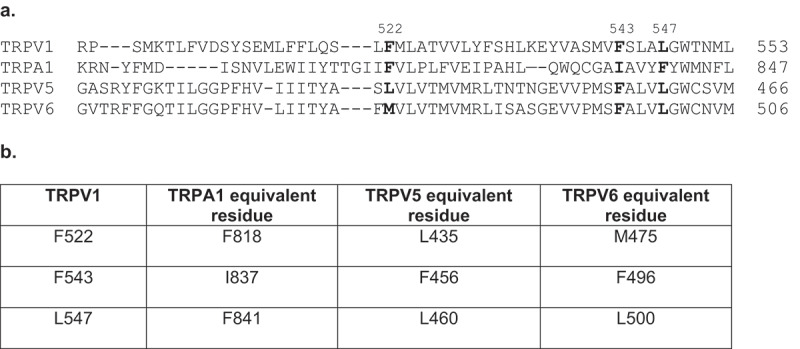
**(a)** Amino acid sequence alignments of TRPA1, TRPV1, TRPV5, and TRPV6, illustrating that the predicted 25OHD-interacting residues within TRPV1 (F522, F543, and L547) are well conserved in the other three TRP channels (bold). (b) Amino acid numbering of the three TRPV1 residues predicted to interact with 25OHD and the equivalent residues in TRPA1, TRPV1, and TRPV6

Our computer modeling of the human TRPV1 predicts that 25OHD binds to TRPV1 in the same vanilloid-binding pocket as the agonist capsaicin and the antagonist capsazepine. However, 25OHD seems to adopt a different binding conformation than either of these two TRPV1 ligands within this binding pocket. These findings suggest that 25OHD may interact with intracellular, not extracellular, binding residues. As the pH sensitivity of TRPV1 is determined by extracellular binding sites, our modeling results may help explain why 25OHD does not modulate low-pH–induced TRPV1 currents, as is the case with anandamide. With respect to the binding location for 25OHD, our modeling predicts that 25OHD binds in the upper region of the vanilloid-binding pocket perpendicular to the binding sites for capsaicin and capsazepine. 25OHD is positioned parallel to the S4-S5 linker, whereas capsaicin and capsazepine are positioned perpendicular to the S4-S5 linker. The previously published cryoEM structure of TRPV1 indicates that the vanillyl ring and amide bonds of capsaicin exhibit van der Waals interactions and hydrogen bonding with the T511, S512, T550, and E570 residues. The flexible acyl tail of capsaicin also interacts with F543, M547, F587, and L669 through van der Waals force [91,105]. These same interacting residues are involved in the binding of resiniferatoxin, another full agonist of TRPV1 [91]. Although the TRPV1 antagonist capsazepine possesses more complex aromatic groups next to its aryl ring in the head region, its amide neck and lipophilic acyl tail regions adopt the same configuration as capsaicin, with capsazepine side-chain atoms interacting with the same TRPV1 residues involved in capsaicin-binding site. In contrast to capsaicin and capsazepine, 25OHD possesses cyclohexane and cyclopentane rings with a hydroxyl group at the end of each molecule (Figure 1). 25OHD also does not possess the planar aromatic ring in the head region and an amide group in the neck region in the structure of capsaicin. Instead, the chair conformation rings of 25OHD likely result in loss of rigidity and also weaken any interactions with the same binding residues as full agonists of TRPV1. However, this lack of rigidity is predicted to allow 25OHD to move more freely within the vanilloid-binding pocket, such that the head and tail of 25OHD reside in the upper portion of the pocket. The 25OHD/TRPV1 *in silico* model predicts that 25OHD interacts with F522, F543, and L547 (Figure 2). Interestingly, the known capsaicin-binding sites F543 and L547, but not Y511, S512, T550, or E570, were predicted in the list of the proposed interacting residues in the 25OHD/TRPV1 model, further supporting the notion that 25OHD resides in the vanilloid-binding pocket, but not precisely at the same location as capsaicin and capsazepine. This unique property of 25OHD may allow 25OHD to weakly activate TRPV1 yet interfere with capsaicin binding within the vanilloid-binding pocket and explain the effects of 25OHD as a TRPV1 partial agonist.

In our recent study, we further investigated the relationship between 25OHD and capsaicin binding at TRPV1 by generating alanine point substitutions at predicted capsaicin-interacting residues Y511, S512, T550, L553, and E570 residues. Consistent with previous studies, all of these alanine mutants, except for L553, significantly reduced TRPV1 capsaicin sensitivity [91,105]. Interestingly, only the Y511A and S512A substitutions abolished the weak stimulatory effect of 25OHD on TRPV1. Overall, these results are in good agreement with the predicted binding of 25OHD within the vanilloid-binding pocket, but not at the same location as capsaicin and capsazepine. Due to the flexible nature of the 25OHD molecule, additional interacting residues likely exist as the *in silico* modeling cannot take into account the full range of binding confirmations possible with such a flexible ligand. Therefore, further structure–function studies are necessary to fully characterize and map the precise residues participating in 25OHD binding to TRPV1.

## Vitamin D regulates PKC-mediated potentiation of TRPV1

TRPV1 activity is potentiated through phosphorylation by PKC, with residues S502 and S800 being identified as the major phospho-acceptor sites within TRPV1 [106–108]. We also demonstrated that 25OHD attenuates the PKC-mediated potentiation of capsaicin-induced TRPV1 current by significantly reducing TRPV1 open probability. Furthermore, we identified the S502, but not S800, as the phospho-acceptor residue playing a key role in this effect of 25OHD. Our *in silico* model predicts that S502 is located near the entrance of the capsaicin-binding pocket and in close proximity to the Y511 and S512 residues that are important in mediating the effects of 25OHD on TRPV1. It is suggested that these three residues may play a role in guiding 25OHD toward its binding sites, i.e., 25OHD may require all three residues as initial contact points to induce a conformational change in TRPV1, allowing 25OHD to reach its optimal position within the binding pocket.

## Does vitamin D regulate other TRP channels?

Our findings on TRPV1 provide direct evidence that vitamin D is capable of regulating calcium homeostasis via a novel mechanism in addition to its actions on the well-documented canonical nuclear VDR pathway. These results also raise the intriguing possibility that vitamin D may also regulate other TRP family members. For example, TRPV6 is crucial for active calcium transport in the intestine [109,110] and 1,25OHD has been shown to alter TRPV6 expression in gut epithelial cells [111]. Interestingly, the overexpression of TRPV6 in VDR knockout mice maintains calcium absorption, indicating that calcium absorption through TRPV6 can occur independently of the VDR [112]. Furthermore, TRPV5 is responsible for calcium uptake in the kidneys, and its expression level is also influenced by 1,25OHD levels [113]. TRPV5 and TRPV6 also possess high selectivity for calcium over sodium ions, with a permeability ratio (P) of P_Ca_>P_Na_ of ~100, while others TRP channels display more selectivity for sodium over calcium ions [114,115]. As the activity of TRPV5 and TRPV6 are known to be modulated by a variety of lipophilic ligands, it is plausible that vitamin D may also regulate the activity of these two TRP family members. Therefore, further investigation into this possibility is warranted as it may shed new light on the mechanisms by which this important vitamin elicits its biological effects. Although TRPV1 is involved in a multitude of cellular pathways, it has been shown that TRPV1 knockout mice exhibit a relatively normal phenotype compared to wild-type mice, suggesting that other TRP family members may be present and compensate for the loss of TRPV1 [116–118]. For example, TRPA1 is thought to act in concert with TRPV1, with both channel types being expressed in a majority of sensory nerves as well as other tissue types in the human body [81,119–122]. Given that TRPA1 is also regulated by lipophilic ligands, this TRP family member may also be regulated by vitamin D.

**Figure 3.**
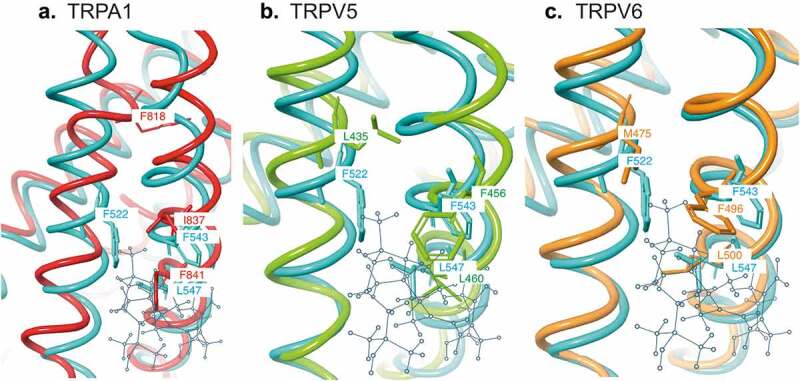
*In silico* homology modeling comparing the structure and 25OHD-interacting residues in TRPV1 with the equivalent residues (Figure 2) and structures of TRPA1 (a), TRPV5 (b), and TRPV6 (c). TRPV1 (light blue [16]), TRPA1 (red, PDB# 6V9W), TRPV5 (light green, PDB# 6B5V), and TRPV6 (orange, PDB# 62EF). The predicted binding site location for the 25OHD structure (gray ball and stick) within TRPV1 [16] is also included for reference

Our docking simulations predict a weak interaction of 25OHD within the vanilloid-binding pocket of TRPV1 due to the flexibility of the 25OHD structure. This apparent flexible nature of the atomistic interaction between 25OHD and TRPV1 provides additional evidence supporting the concept that vitamin D may regulate additional TRP family members’ activity. This flexibility of binding is in direct contrast to the higher affinity interaction of TRPV1 with other TRPV1-specific ligands such as capsaicin that are more rigid in their structure. If this is indeed the case, then vitamin D has the potential to be pharmacologically promiscuous, also modulating the activity of other TRP family members. This notion is further supported by the analysis of the amino acid sequences of the above mentioned TRP channels, TRPA1, TRPV1, TRPV5, and TRPV6, where three of the predicted 25OHD interacting residues within TRPV1 (F522, F543 and L547) are either the same or possess similar side-chain properties to the equivalent residues in these other TRP channels (Figure 2). Furthermore, we performed *in silico* homology modeling to compare the putative vitamin D–binding pocket structure within TRPV1 with the structures of TRPA1, TRPV5, and TRPV6. This modeling revealed that there is a very good agreement for the location of these predicted interacting residues within the structure of all four TRP channels (Figure 3). Taken together, these results support the notion that vitamin D may also modulate the activity of other TRP family members, and future studies are warranted to further investigate this fascinating possibility.

## Conclusions

The major vitamin D metabolites such as 25OHD and 1,25OHD should now be added to the list of known endogenous ligands for TRPV1. This observation may have important implications for fully understanding the biological actions of this vitamin in the human body. On its own, vitamin D may act as a partial TRPV1 agonist that may promote a small but sustained calcium influx into cells without initiating calcium-induced desensitization. Conversely, vitamin D can also act as an antagonist in the presence of a full agonist, therefore decreasing TRPV1-mediated calcium influx and may act to oppose excessive calcium influx and over-activation of calcium-dependent cellular signaling. This dual function of vitamin D on TRPV1 activity may provide an elegant means to not only generate a tonic basal calcium signal but also prevent over-activation or calcium-induced damage in the presence of a full agonist. Future research on this topic will contribute to a greater understanding of diseases where vitamin D deficiency and TRPV1 activity have been associated, such as chronic pain [87,123], and autoimmune diseases including multiple sclerosis and type 1 diabetes [11,66,75,82,124].
